# Sexual dimorphism in adipose tissue mitochondrial function and metabolic flexibility in obesity

**DOI:** 10.1038/s41366-021-00843-0

**Published:** 2021-05-17

**Authors:** Amanda D. V. MacCannell, T. Simon Futers, Anna Whitehead, Amy Moran, Klaus K. Witte, Lee D. Roberts

**Affiliations:** Leeds Institute of Cardiovascular and Metabolic Medicine, University of Leeds, Leeds, LS29JT UK

**Keywords:** Fat metabolism, Energy metabolism, Obesity, Homeostasis

## Abstract

**Objective:**

The prevalence of obesity is growing globally. Adiposity increases the risk for metabolic syndrome, type 2 diabetes and cardiovascular disease. Adipose tissue distribution influences systemic metabolism and impacts metabolic disease risk. The link between sexual dimorphisms of adiposity and metabolism is poorly defined. We hypothesise that depot-specific adipose tissue mitochondrial function contributes to the sexual dimorphism of metabolic flexibility in obesity.

**Methods:**

Male and female mice fed high fat diet (HFD) or standard diet (STD) from 8–18 weeks of age underwent whole animal calorimetry and high-resolution mitochondrial respirometry analysis on adipose tissue depots. To determine translatability we used RT-qPCR to examine key brown adipocyte-associated gene expression: peroxisome proliferator-activated receptor co-activator 1α, Uncoupling protein 1 and cell death inducing DFFA like effector a in brown adipose tissue (BAT) and subcutaneous adipose tissue (sWAT) of 18-week-old mice and sWAT from human volunteers.

**Results:**

Male mice exhibited greater weight gain compared to female mice when challenged with HFD. Relative to increased body mass, the adipose to body weight ratio for BAT and sWAT depots was increased in HFD-fed males compared to female HFD-fed mice. Oxygen consumption, energy expenditure, respiratory exchange ratio and food consumption did not differ between males and females fed HFD. BAT mitochondria from obese females showed increased Complex I & II respiration and maximal respiration compared to lean females whereas obese males did not exhibit adaptive mitochondrial BAT respiration. Sexual dimorphism in BAT-associated gene expression in sWAT was also associated with Body Mass Index in humans.

**Conclusions:**

We show that sexual dimorphism of weight gain is reflected in mitochondrial respiration analysis. Female mice have increased metabolic flexibility to adapt to changes in energy intake by regulating energy expenditure through increased complex II and maximal mitochondrial respiration within BAT when HFD challenged and increased proton leak in sWAT mitochondria.

## Introduction

The prevalence of obesity, defined by a BMI of ≥30 and characterised by excess adiposity, is growing globally. Obesity perturbs numerous metabolic pathways [1] and increases the risk for metabolic syndrome, a combination of high waist circumference, circulating levels of triacylglycerols (TAGs) and low-density lipoprotein cholesterol, fasting hyperglycaemia and high blood pressure [2]. In turn, metabolic syndrome increases the risk of developing type 2 diabetes and cardiovascular disease [3].

Adipose tissue depots exhibit distinct molecular and functional characteristics. These depots vary in size, ranging from 5% to 60% of total body weight [4]. White adipose tissue (WAT) is the primary metabolic energy reserve, it synthesises and stores TAG when caloric consumption exceeds expenditure. There are two primary and distinct locations of WAT within mammals: subcutaneous (sWAT) and visceral (vWAT). Distribution of WAT influences the risk of metabolic disease [5]. vWAT is located within the abdominal cavity [6] and is associated with a higher risk of metabolic disease and type 2 diabetes in both men and women [7, 8]. sWAT is a subdermal depot and its role in metabolic disease is still poorly defined [9]. In addition, brown adipose tissue (BAT) is primarily located in the interscapular region of mice, and the neck in humans, and functions to generate heat, in a process known as non-shivering thermogenesis, in response to cold [10]. BAT converts fatty acids into measurable changes in heat production through the futile cycling of the electron transport chain (ETC) mediated by Uncoupling Protein 1 (UCP1) [11]. Evidence suggests active BAT may protect against obesity and cardiometabolic disease [12]. Distribution of the three adipose depots confer varying risks for metabolic disorders [5].

There are fundamental sex-specific differences in adipose tissue distribution and adiposity, which may translate to differences in the prevalence of metabolic syndrome and type 2 diabetes [13, 14]. Differences in waist circumference between sexes has been linked to increased cardiovascular risk [15]. Although women have ~10% higher body fat compared to men [16], premenopausal women have fewer incidents of obesity related metabolic disorders than men [17]. Adipose distribution in women is characterised by more sWAT and less vWAT compared with men [18, 19]. Fundamental systemic metabolic characteristics exhibit sexual dimorphism. Women have lower absolute energy expenditure compared with men [20, 21] and men have higher fasting glucose concentrations [22]. In mouse studies male mice rapidly increase body mass when high fat challenged [23], but the mechanisms for the sex differences in weight gain are unknown [24]. Here, we define key metabolic differences between the sexes which may contribute to the resistance to rapid weight gain observed in female mice. We find novel sex-specific differences in the mitochondrial metabolism and function of BAT, sWAT and vWAT in mice in response to a high fat feeding challenge.

## Materials and methods

### Animals and diets

All experiments and procedures involving live animals were performed in accordance with Home Office UK regulation - Animals (Scientific Procedures) Act 1986. Mice were bred from C57BL6/N mice at the University of Leeds. Animals had access to food and water ab libitum on a controlled 12-h light/dark cycle in a pathogen free facility. Mice were group housed in cages of five with a dome. All mice were fed standard diet (STD) (Special Diet Services 8072; 3.592 kcal/g) up to 8 weeks of age, at 8 weeks half of the mice were randomly assigned to high fat diet (HFD) 60% fat diet (F3282, Datesand Ltd, Manchester UK; 5.49 kcal/g) for 10 weeks.

### Comprehensive lab animal monitoring system (CLAMS)

Whole animal calorimetry was performed by singly housing mice in a Comprehensive Laboratory Animal Monitoring System (CLAMS; Columbus Instruments). All mice had a minimum acclimation period of 72 h before experimental recording began for 24 h. Within CLAMS oxygen consumption, carbon dioxide production, respiratory exchange ratio (RER), food intake and activity were recorded.

### High-resolution tissue respirometry

Mice were sacrificed using cervical dislocation. Interscapular BAT (iBAT), subcutaneous inguinal WAT (iWAT) and visceral epididymal WAT (eWAT) were placed into biopsy preservation solution for high-resolution respirometry (BIOPS) [25]. After dissection of both right and left iBAT, iWAT and eWAT pads under a dissecting microscope, tissue was pat dried and weighed. Tissue was permeabilized with freshly prepared saponin (50 μg mL^−1^) and washed in mitochondrial respiration medium, MiR05 [26]. Using Oxygraph-O2k high resolution respirometry (OROBOROS) we measured respiration in tissue slices of the three adipose depots. Complex I-mediated respiration was measured with glutamate (10 mM) and malate (1 mM) and then again in the presence of ADP (2.5 mM). Cytochrome c (Cytc; 10 μM) ensured saponin permeabilization did not affect the integrity of the outer mitochondrial membrane. The addition of succinate (10 mM) provided a measure of complex I and II-supported oxidative phosphorylation. In BAT and sWAT guanosine 5′-diphosphate sodium salt (GDP) was titrated in 1 mM at a time to determine respiration rates not contributed to by UCP1. GDP titration was not assessed in eWAT due to low UCP1 expression in this tissue. Maximal ETC capacity was assessed with a bolus of carbonyl cyanide *m*-chlorophenyl hydrazine (CCCP; 5 μM in DMSO). The addition of rotenone (0.5 μM), a complex I inhibitor, gave complex II-supported oxidative phosphorylation in isolation. Finally, antimycin A (12.5 μM), provided an estimate of non-mitochondrial (background) respiration that was subtracted as a correcting factor from all respiratory measurements. The respiratory control ratio (RCR) was calculated by dividing the respiration rate following ADP addition over the rate prior to ADP addition [27, 28].

### Gene expression analysis

Total RNA extraction from iBAT and iWAT; cDNA conversion; and quantitative RT-PCR were performed according to published protocols [29]. All data were normalised to 18S rRNA and quantitative measures were obtained using the ΔΔCT method.

### Human adipose tissue

Fifty patients undergoing routine de-novo pacemaker implantation at Leeds General Infirmary volunteered to participate in the study and provided written consent (Supplementary Table 1). Human sWAT biopsies were obtained prior to any instrumentation of the central circulation. Lidocaine was initially injected to anaesthetise the area and a small incision was made under the left clavicle. Immediately prior to creation of the pre-pectoral pocket for the generator above the pectoralis major, a portion of sWAT (~100 mg) was sampled and immediately snap frozen in liquid nitrogen. Following this, pacemaker procedures were otherwise completed routinely. There were no complications ascribed to the sampling procedure. The study is approved by the Leeds West Research Ethics Committee (11/YH/0291) and Leeds Teaching Hospitals Trust R&D committee (CD11/10015) and conforms to the Declaration of Helsinki.

### Statistical analysis

Mass, respirometry and mRNA data were analysed using Prism 8 (version 8.3.0). Differences between groups were compared for statistical significance by ANOVA or two-tailed Student’s *t* test. CLAMS data analysis was performed using CalR [30]. ANCOVA with mass as a dependent variable was used to compare oxygen consumption, carbon dioxide production and energy expenditure. Linear regression using Pearson correlation was used when analysing the human mRNA data. Group variance was analysed with an *F*-test. Sample size was determined by power calculation. Data are expressed as mean ± standard error of the mean, *P* < 0.05 denoted significance.

## Results

### Male mice have increased body mass and impaired metabolic function compared to female mice when high fat diet challenged

**Fig. 1:**
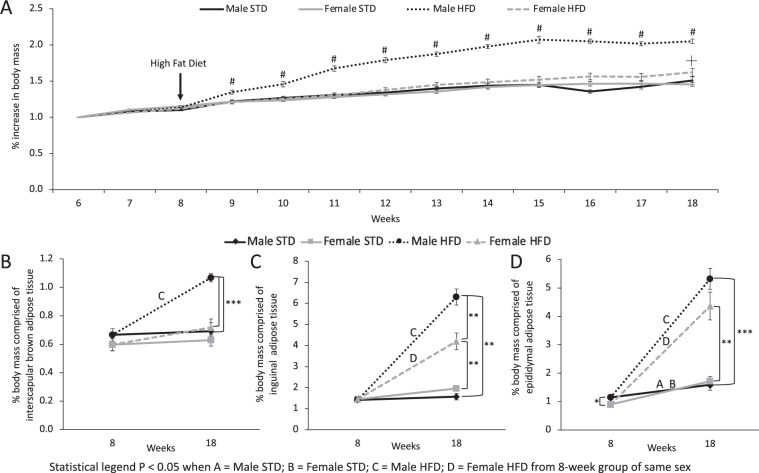
Male mice fed high fat diet have increased body mass and adipose tissue depot mass. **A** Body mass (BM) increase from 6 to 18 weeks of age in male and female mice. Arrow indicates high fat diet (HFD) feeding at 8 weeks of age. # indicates the timepoint when male HFD mice body weight is significantly greater than all other groups (*P* < 0.05). ┼ indicates when female HFD mice are significantly different than all other groups (*P* < 0.05). There is a significant effect of time (*F*_(4.4, 161.4)_ = 302, *P* < 0.0001), group (*F*_(3.0, 38)_ = 69.5, *P* < 0.0001) and interaction between time and group (*F*_(36, 440)_ = 22.2, *P* < 0.0001) on the percent increase in body mass. Percentage of body mass comprised of **B** iBAT, **C** iWAT and **D** eWAT of male and female mice fed either standard diet (STD) or HFD for 10 weeks. Data are presented as mean ± SEM, 8-weeks-old males (*n* = 10), 8-week-old females (*n* = 9), Male STD (*n* = 10), Female STD (*n* = 9), Male HFD (*n* = 10), Female HFD (*n* = 10). *P < 0.05, **P < 0.01, ***P < 0.0001. “A” for 8-week-old males compared to Male STD; **B** 8-week-old females compared to Female STD; **C** 8-week-old males compared to Male HFD; **D** 8-week-old females compared female HFD.

We fed male and female mice HFD for 10 weeks, a commonly used model of obesity and insulin resistance [31]. There is no difference in the percent increase in body mass between the sexes before diet intervention (Fig. 1A). After 1 week of HFD feeding male mice had significantly increased body mass compared with STD fed male mice. Female mice fed HFD did not exhibit an increase in body mass compared with STD controls until 9 weeks of HFD feeding (Fig. 1A).

Eight-week-old male and female mice exhibit no significant difference in the percentage of body mass comprised of iBAT (Fig. 1B). In both sexes STD feeding does not significantly affect iBAT depot mass between 8 weeks and 18 weeks old (Fig. 1B). HFD increased iBAT mass in male mice compared to 8-week-old baseline mice and STD fed 18-week old male mice, this effect was not observed in female mice (Fig. 1B). Similar to iBAT, there was no significant difference in iWAT mass between 8 week old male and female mice (Fig. 1C). In both sexes STD feeding from 8 weeks to 18 weeks does not significantly affect iWAT depot mass (Fig. 1C). However, HFD feeding significantly increases percent body mass comprised of iWAT in both sexes (Fig. 1C). Male mice fed HFD have iWAT depots significantly greater in mass than iWAT depots from female mice fed HFD (Fig. 1C). Eight-week-old male mice had significantly higher eWAT mass compared to female mice (Fig. 1D). After 10 weeks of STD feeding both sexes had greater eWAT mass compared to 8 weeks of age (Fig. 1D). The increase in eWAT mass is further enhanced in both sexes by HFD feeding for 10 weeks (Fig. 1D).

Therefore, male mice fed HFD rapidly gain weight, in part through increased adipose tissue depot mass, compared to female mice. Increased body and adipose tissue mass corresponds to impaired glucose tolerance in male HFD fed mice (Supplementary Fig. 1). Increased adipose mass in males but not females could contribute to sex-specific glucose intolerance induced by HFD.

### Age and diet have inverse effects on whole animal metabolic phenotype between sexes

**Fig. 2:**
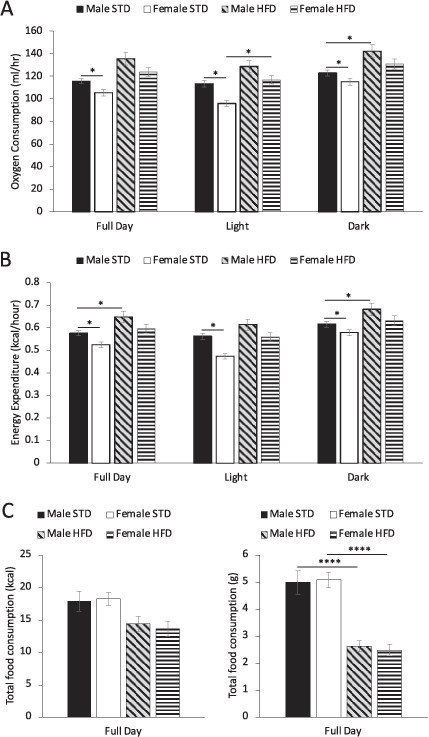
Metabolic phenotypic effect of 10 weeks standard chow (STD) or high fat diet (HFD) feeding on male and female mice. **A** Oxygen consumption, **B** energy expenditure and **C** energy (kcal) and grams of total food consumption in a 24-h period. Data are presented as mean ± SEM, Male STD (*n* = 8), Female STD (*n* = 9), Male HFD (*n* = 10), Female HFD (*n* = 10). Data are presented as mean ± SEM, differences were considered to be statistically significant at *P < 0.05, **P < 0.01, ***P < 0.001.

Due to the rapid weight gain in male mice, we used whole body calorimetry to characterise the effects of HFD on systemic energy expenditure (Supplementary Table 2). Male mice fed STD have higher oxygen consumption and energy expenditure throughout a 24 h period compared to female STD fed mice (Fig. 2A, B). When fed HFD, males increase energy expenditure compared to STD fed male mice (Fig. 2B). HFD feeding of females did not affect energy expenditure when compared to STD chow fed mice (Fig. 2B). Ten weeks of STD feeding in female mice did not affect oxygen consumption or energy expenditure compared with 8 week baseline mice (Supplementary Fig. 2A, B). However energy expenditure and oxygen composition increases in male mice fed STD by 18 weeks compared to 8 week old male mice (Supplementary Fig. 2A, B).

Male and female mice have lower RER when fed HFD compared to their STD fed controls (Supplementary Fig. 3C). Both male and female mice fed HFD for 18 weeks had significantly reduced food intake in terms of food weight compared with STD fed animals (Fig. 2C). However due to the calorie-dense nature of the HFD, 18-week-old mice regardless of diet and sex, exhibit no difference in the total energy content (kcal) of food consumed (Fig. 2C).

Therefore, male mice have higher energy expenditure than female mice when fed STD and further increased energy expenditure when fed HFD. Both sexes fed HFD preferentially oxidise lipid as a fuel source and there is no difference in total food consumption regardless of diet or sex at 18 weeks of age. Although, there are behavioural changes resulting in decreased locomotor activity observed only in HFD fed male mice but not females (Supplementary Fig. 3A, B), this did not affect energy expenditure. These data indicate that the extreme weight gain phenotype in male mice fed HFD is not due to systemic metabolic differences.

### Both diet and sex influence respiration of adipose tissue mitochondria

To investigate potential underlying mechanisms for sexual dimorphisms of adipose depot size and whole-body weight gain, we used high-resolution respirometry to investigate depot-specific adipose tissue mitochondrial function in iBAT, sWAT and eWAT.

**Fig. 3:**
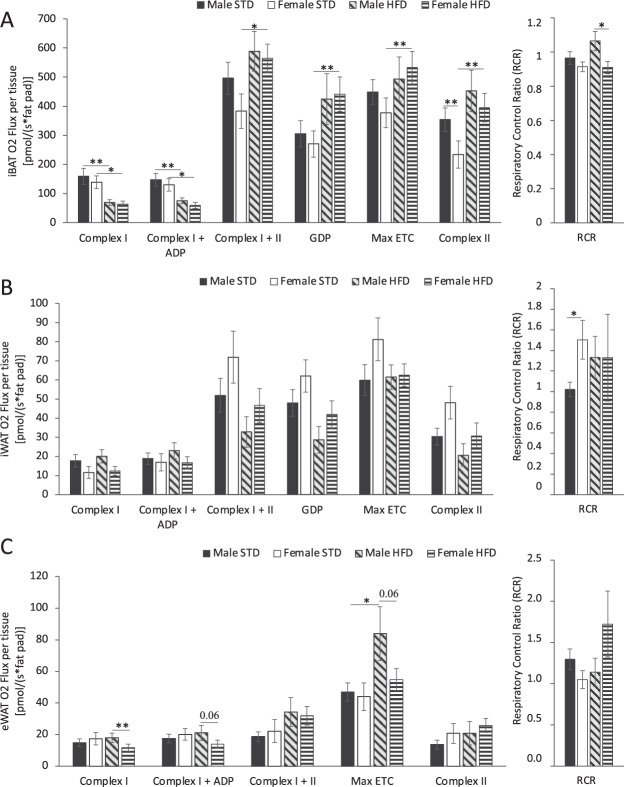
The effect of 10 weeks standard chow diet (STD) or high fat diet (HFD) feeding on adipose tissue mitochondrial respiration in male and female mice. Respiration stimulated with Complex I (glutamate and malate), Complex I ADP (ADP) Complex II (succinate) substrates, inhibition of UCP1 with GDP, maximally uncoupled electron transport chain (ETC) (FCCP) and respiratory control ratio (RCR) of permeabilized **A** iBAT, **B** iWAT and **C** eWAT of male and female mice fed either STD or HFD. Data are presented as mean ± SEM, Male STD (*n* = 10), Female STD (*n* = 9), Male HFD (*n* = 10), Female HFD (*n* = 10). Data are presented as mean ± SEM, differences were considered to be statistically significant at *P < 0.05, **P < 0.01.

Eight week old male and female mice showed no significant difference in mitochondrial respiration at complexes I and II in iBAT (Supplementary Fig. 4A). iBAT of male and female mice exhibited reduced flux through complex I independent of ADP stimulation, in response to HFD (Fig. 3A). BAT of female mice fed HFD exhibited greater respiration when complexes I and II were stimulated together, Ucp1 was inhibited with GDP, and when maximum uncoupled respiration and complex II flux in isolation were analysed compared to STD fed female mice. iBAT of Male mice did not exhibit this enhanced mitochondrial respiration response when challenged by HFD (Fig. 3A). Comparing complex I and II mitochondrial respiration in iBAT between 8-week-old mice and 18-week-old mice, there is a significant increase in mitochondrial respiration when both sexes were fed HFD (Supplementary Fig. 4A). When GDP is used to inhibit Ucp1 there is no sex-specific difference in the respiration of iBAT regardless of time and diet (Supplementary Fig. 4A). Mitochondrial respiration of iBAT from female mice fed HFD was significantly higher than respiration in iBAT from STD fed female mice when Ucp1 is inhibited with GDP. There is no significant difference in GDP inhibited respiration between male mice fed HFD and STD (Fig. 3A). RCR is used to measure the coupling efficiency of the ETC in the formation of ATP. In iBAT the RCR of male mice fed HFD increased compared to 8-week-old mice (Supplementary Fig. 4A). In contrast, Female mice do not exhibit this change in RCR in response to HFD challenge, as time and diet do not influence the RCR of female mice (Supplementary Fig. 4A; Fig. 3A).

In iWAT, sex, diet and age did not affect flux through mitochondrial ETC complexes (Fig. 3B; Supplementary Fig. 3B). However, iWAT from female STD fed mice has a higher RCR than iWAT from male STD fed mice (Fig. 3B). This indicates increased coupling efficiency of the ETC in the formation of ATP in female mice compared to male mice under STD conditions (Fig. 3B). Mitochondrial respiration in eWAT did not exhibit sexual dimorphism in mice fed STD (Fig. 3C). However, eWAT from male mice fed HFD had increased flux through complex I compared to female mice fed HFD (Fig. 3C). Male mice had significantly increased eWAT complex I and II respiration from 8-weeks to 18-weeks of age with both STD and HFD (Supplementary Fig. 4C). eWAT in 8-week-old female mice had increased coupling efficiency as determined by RCR compared to male 8-week-old mice, however this sexual dimorphism is not maintained by 18 weeks (Supplementary Fig. 3C).

These results indicate that HFD feeding impairs flux through the beginning of the electron transport chain (complex I) in the iBAT of both sexes. However, in response to HFD, female mice exhibit enhanced respiration throughout the ETC of iBAT (complex II, GDP, maximum respiration). An effect not observed in male mice in response to HFD challenge. A lower proportion of iBAT respiration in female mice is also accounted for by Ucp1 (GDP inhibited respiration). Diet, sex and time does not affect respiration in iWAT. However male mice have increased mitochondrial respiration in eWAT compared with females in response to HFD challenge.

### Sex-specific differences in adipose tissue mitochondrial function correspond to the expression of brown adipose-associated genes

**Fig. 4:**
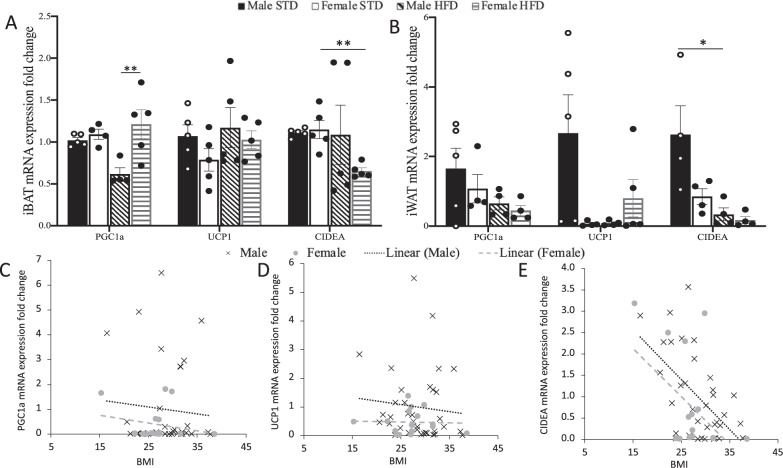
Sex and diet influence thermogenic gene expression in mice and humans. Gene expression of *Pgc1a*, *Ucp1* and *Cidea* in **A** BAT and **B** sWAT of male and female mice fed HFD or STD for 10 weeks. Data are presented as mean ± SEM, differences were considered to be statistically significant at *P < 0.05, **P < 0.01. Human gene expression of **C** *PGC1α*, **D** *UCP1* and **E** *CIDEA* correlated to BMI. Human females *n* = 16, human males *n* = 34. Expression of *PGC1α* and *UCP1* slope of both males and females is not significantly different from zero or each other. Slope of CIDEA expression in males is significantly different from zero (*P* = 0.0004).

Sex-specific differences in the Ucp1 contribution to mitochondrial respiration were further evaluated by examining expression of key brown adipocyte-associated genes. We used RT-qPCR to analyse the expression of brown adipocyte-associated genes: peroxisome proliferator-activated receptor co-activator 1α (*Pgc1α*), *Ucp1* and cell death inducing DFFA like effector a (*Cidea*) in iBAT and iWAT of 18-week-old mice. HFD-induced obesity decreases *Pgc1α* expression in iBAT of male mice, female mice were resistant to this effect (Fig. 4A). Sex and diet did not affect *Pgc1α* expression in iWAT (Fig. 4B). In both iBAT and iWAT *Ucp1* expression is not significantly different between sex or diet (Fig. 4A, B). *Cidea* expression is decreased in the iBAT of female mice fed HFD compared with females fed STD. Whereas the expression of *Cidea* in males fed HFD is unchanged when compared with male mice fed STD (Fig. 4A). Contrastingly, expression of *Cidea* in the iWAT of male mice fed HFD was significantly lower than in the iWAT of STD fed male mice. There was no significant effect of diet on *Cidea* expression in the iWAT of female mice.

To determine if gene expression differences in mice were reflected in humans, we correlated expression of *PGC1α*, *UCP1* and *CIDEA* in sWAT of fifty human volunteers to BMI. *PGC1α* and *UCP1* gene expression is not correlated to BMI in our volunteers and there is no difference between sexes (Fig. 4C, D). However, *CIDEA* gene expression in sWAT of human males is significantly inversely correlated with BMI, this effect is not observed in females (Fig. 4E). Consistent with the gene expression of mice, sWAT *CIDEA* expression is negatively correlated with body mass in male humans and significantly decreased in male mice fed HFD.

## Discussion

The aim of this study was to examine sex-specific differences in systemic metabolism and adipose depot mitochondrial function in response to high fat diet-challenge and obesity. We began this study by validating previously reported observations detailing enhanced weight gain and decreased glucose tolerance and insulin sensitivity in male mice challenged with HFD compared to female mice [23, 32, 33]. The rapid weight gain in male HFD fed mice was not explained by differences in food consumption, energy expenditure or activity alone. Although increased activity in female mice could contribute to increased metabolic flexibility [34], the whole animal metabolic parameters did not explain the differences in body mass.

To establish whether sexual dimorphism in fat distribution contributes to differences in body mass we examined three key adipose depots, iBAT, iWAT and eWAT. Interestingly, at 8 weeks of age, and prior to HFD challenge, male mice had significantly greater eWAT mass compared to female mice. Future studies may focus on investigating whether increased basal eWAT mass contributes to the enhanced weight gain observed in male mice in response to HFD challenge possibly through techniques such as omentectomy. The differences in body mass as a result of HFD feeding are reflected in adipose depot mass with increased iBAT, iWAT and eWAT in HFD fed males and iWAT and eWAT in HFD fed females. Female iBAT was resistant to the depot size increase observed in male mice fed HFD.

Differences in adiposity and mitochondrial function between males and females are likely due to the contribution of sex steroid in the regulation of adipocyte development, function and lipid retention, which appears to be negatively regulated by oestrogen and can be explained by oestrogen mediated lipid accumulation [35, 36]. Future studies using a neutered mouse model would facilitate further investigation of the contribution of sex steroid hormones to the differences in adipose depot size, weight gain, and mitochondrial function between male and female mice in the setting of high fat feeding. Although, with the caveat that negation of sex steroid hormone effects will alter other physiological parameters influencing weight gain including basal metabolic rate, organismal growth and lean muscle mass.

Age influenced the adiposity of WAT in the same manner for both sexes. However, only male mice fed HFD had increased BAT depot mass, a phenomenon previously observed in response to HFD [37]. This may be due to increased whitening of the BAT depot in male mice as the increased BAT did not reflect in increased whole animal energy expenditure, reduced weight gain or BAT metabolic mitochondrial function.

Both the distribution and amount of adipose tissue have consequences for metabolism and metabolic health. We observed differences in oxygen consumption, energy expenditure and RER for both sexes and diets with age, this may be due to changes in lean body mass as the mice age, which has also been observed in humans [38]. Female mice fed STD have significantly lower oxygen consumption and energy expenditure than male mice fed STD. With HFD feeding this sex difference is lost. This suggests that the lower baseline energy expenditure and oxygen consumption in female mice provides females greater metabolic flexibility to adapt to HFD challenge compared to males. Given the central role of mitochondria in energy metabolism, measuring oxygen consumption directly at the mitochondrial level provides information that cannot be determined systemically. Therefore, we used high-resolution respirometry to determine mitochondrial function in the adipose tissue depots of obese and lean male and female mice. Female mice fed HFD have significantly increased mitochondrial iBAT respiration compared to STD fed female mice, with succinate as a substrate. This difference is maintained after Ucp1 is inhibited with GDP. Therefore Ucp1 is unlikely to contribute to the increased iBAT respiration in female mice fed HFD. This is confirmed by the increase in maximum respiration when the iBAT mitochondria were uncoupled in HFD fed female mice compared to STD fed female mice. Together these results indicate that HFD feeding impairs flux through the beginning of the electron transport chain (complex I) in the iBAT equally in both sexes. However, the iBAT of female mice fed HFD adapts to this challenge with enhanced respiration through complex II and maximum ETC respiration. The BAT of male mice does not similarly respond to HFD challenge. This phenomenon may be reflected systemically. STD chow fed male mice exhibit greater basal energy expenditure compared to female mice fed STD. This sex difference in whole body energy expenditure is abolished when the animals are challenged by HFD. These findings suggest that female mice have a greater latent capacity for metabolic flexibility in BAT mitochondrial function in response to obesity compared to male mice and this may translate to effects on systemic energy expenditure.

Both the iWAT and eWAT do not show strong phenotypic differences in mitochondrial respiration between males and females. However, iWAT from female STD fed mice have a higher RCR than iWAT from male STD fed mice suggesting greater ETC coupling efficiency. Although, this sex-specific difference is abolished by HFD feeding. The increase in body mass, adipose tissue depot size and decreased BAT mitochondrial metabolism of HFD fed male mice compared to HFD fed female mice indicates that female mice have increased metabolic flexibility to adapt to changes in energy intake by regulating energy expenditure through adaptive respiration in BAT mitochondria. Increased RCR in iWAT of female mice on STD also suggests a greater metabolic flexibility and range to adapt to energetic challenge.

Using both sexes of mice increases the translatability of laboratory research into the clinical setting. When comparing mRNA expression of *Pgc1α*, *Ucp1* and *Cidea* of iWAT from mice and humans we show that mRNA expression of our mouse model is reflected in human volunteers. *PGC1α* and *UCP1* show no difference in expression between diet in mice or correlation with BMI in humans. However, *CIDEA* mRNA expression in male humans is negatively correlated with BMI. This is comparable to the decreased *Cidea* expression in male mice fed HFD compared to STD. Female mice and humans do not have significantly decreased *CIDEA* expression with HFD feeding and increasing BMI. Decreased *Cidea* expression in sWAT has been previously shown to inhibit the uncoupling of BAT respiration in mice [39]. Cidea regulates metabolism through Ucp1 [40] and increased *Cidea* expression is correlated with lipid droplet size in sWAT [41], which could contribute to the increased sWAT observed in male mice fed HFD compared to female mice fed HFD. Therefore, the significant decrease in *Cidea* when male mice are fed HFD could explain why, although male mice have a significantly greater amount of sWAT, this is not translated to increased respiration in the mitochondria, especially with GDP mediated Ucp1-inhibition.

Here, we investigate the relationship between sex and mitochondrial metabolism in adipose tissue. Male mice rapidly gain weight when high fat challenged, and this may be due to sexual dimorphism of BAT mitochondrial respiration. We describe distinct sex-specific differences in the systemic and adipose tissue metabolism of mice in response to high fat challenge and obesity. These findings emphasise the necessity of including both sexes in translational research of metabolic diseases. Moreover, we identify reduced metabolic flexibility in the systemic phenotype and BAT respiration of male mice compared to a greater latent capacity for mitochondrial metabolic flexibility in the BAT of female mice. This sex-specific difference in BAT metabolic flexibility may underlie the increased susceptibility of males to weight gain and an impaired metabolic phenotype in response to obesity.

## Supplementary information

Supplementary Material
