# Supplementary material for: Sexual dimorphism in adipose tissue mitochondrial function and metabolic flexibility in obesity
Source: Int J Obes (Lond). 2021 May 17;45(8):1773–81. doi: 10.1038/s41366-021-00843-0 (PMC8310795; doi:10.1038/s41366-021-00843-0)
Supplement: Supplementary file 1 — Supplementary Material [file 41366_2021_843_MOESM1_ESM.docx]

**SUPPLEMENTARY MATERIAL**

**Sexual dimorphism in adipose tissue mitochondrial function and metabolic flexibility in obesity**

Amanda DV MacCannell^1^, Simon Futers^1^, Anna Whitehead^1^, Amy Moran^1^, Klaus K Witte^1^, Lee D Roberts^1^

1) Leeds Institute of Cardiovascular and Metabolic Medicine, University of Leeds, Leeds LS29JT, UK.

**Supplementary Methods**

**Glucose Tolerance Test (GTT) and Insulin Tolerance Test (ITT)**

Prior to GTT, mice were fasted overnight (16 hours) and blood glucose levels were measured using an On Call Plus II Glucose meter. Mice were given an intra-peritoneal (IP) injection of 1 mg/g of D-glucose (Sigma 49139). Blood glucose concentration was measured every 30 minutes for 2 hours. Prior to ITT, mice were fasted in the morning for 2 hours and fasting blood glucose levels measured. Mice were injected intraperitoneally with insulin (0.75 IU/Kg in PBS). Blood glucose concentrations were measured every 15 minutes for 90 minutes.

**Supplementary Fig. 1 Male mice have impaired insulin and glucose tolerance compared to female mice when fed high fat diet.** **A** Blood glucose (mmol/L) in male and female mice fed standard diet (STD) or high fat diet (HFD) after bolus administration of glucose. **B** Blood glucose (mmol/L) in male and female mice fed STD or HFD after bolus administration of insulin. **C** Glucose tolerance test area under the curve. **D** Insulin tolerance test area under the curve. # indicates when male HFD blood glucose is significantly higher than all other groups (P < 0.05). Male STD (n = 10), Female STD (n = 9), Male HFD (n = 10), Female HFD (n = 10). Data are presented as mean ± SEM. *** P < 0.0001.

**Supplementary Fig. 2 Metabolic phenotypic effect of 10 weeks standard chow (STD) or high fat diet (HFD) feeding on male and female mice. A** Oxygen consumption, **B** Energy Expenditure, **C** Respiratory Exchange Ratio (RER) and **D** total food consumption (kcal), **E** total food consumed in grams in a 24 hr period. Data are presented as mean ± SEM, 8-weeks-old males (n = 10), 8-week-old females (n = 9), Male STD (n = 8), Female STD (n = 7), Male HFD (n = 10), Female HFD (n = 10). Difference were considered to be statistically significant at P < 0.05, difference are denoted as: “A” for 8-weeks-old males compared to Male STD; “B” 8-weeks-old females compared to Female STD; “C” 8-weeks-old males compared to Male HFD; “D” 8-weeks-old females compared female HFD. Statistical analysis was performed using ANCOVA with body weight as a covariate for **A-C** and ANOVA for **D-E**.

**Supplementary Fig. 3 Metabolic phenotyping of STD and HFD fed male and female mice. A** Locomotor Activity, **B** Ambulatory Activity and **C** Respiratory Exchange Ratio of male and female mice fed either STD or HFD for 10 weeks. Data are presented as mean ± SEM, Male STD (n = 8), Female STD (n = 7), Male HFD (n = 10), Female HFD (n = 10). Difference were considered to be statistically significant at P < 0.05, difference are denoted as: “a” for male STD compared to female STD; “b” for Male HFD compared to Female HFD; “c” for Male STD compared to Male HFD; “d” for Female STD compared to F HFD.

**Supplementary Fig. 4 The effect of 10 weeks standard chow (STD) or high fat diet (HFD) feeding on adipose tissue mitochondrial respiration in male and female mice.** State 4 respiration stimulated with Complex I (glutamate and malate), Complex I ADP (ADP) Complex II (succinate) substrates, inhibition of UCP1 with GDP and respiratory control ratio (RCR) of permeabilized **A** brown adipose tissue (BAT), **B** sWAT and **C** vWAT of male and female mice at 8 weeks of age and following 10 weeks of either STD or HFD. GDP rate was not determined in vWAT due to lack of UCP1 present. Data are presented as mean ± SEM, 8-weeks-old males (n = 10), 8-week-old females (n = 9), Male STD (n = 10), Female STD (n = 9), Male HFD (n = 10), Female HFD (n = 10). Difference were considered to be statistically significant at P < 0.05, difference are denoted as: “A” for 8-weeks-old males compared to Male STD; “B” 8-weeks-old females compared to Female STD; “C” 8-weeks-old males compared to Male HFD; “D” 8-weeks-old females compared female HFD.

**Supplementary Table 1. Human Patient Demographics**

| **Samples Number** | **Sex** | **Age** | **Weight (kg)** | **Height (m)** | **BMI** | **BMI Ranking** 1=Normal (< 25); 2=overweight (25-30);  3 = obese (>30) |
| --- | --- | --- | --- | --- | --- | --- |
| 20 | F | 81.3 | 35.4 | 1.52 | 15.3 | 1 |
| 157 | F | 65.8 | 64.4 | 1.70 | 22.3 | 1 |
| 280 | F | 72 | 69.9 | 1.70 | 24.1 | 1 |
| 295 | F | 90 | 69.4 | 1.68 | 24.7 | 1 |
| 305 | F | 75 | 62 | 1.61 | 23.9 | 1 |
| 14 | F | 71.5 | 74.0 | 1.67 | 26.5 | 2 |
| 17 | F | 74.8 | 77.6 | 1.69 | 27.2 | 2 |
| 19 | F | 79.9 | 73.0 | 1.60 | 28.5 | 2 |
| 139 | F | 69.2 | 76.4 | 1.60 | 29.8 | 2 |
| 146 | F | 82.6 | 69.9 | 1.60 | 27.3 | 2 |
| 206 | F | 76 | 67.6 | 1.58 | 27.1 | 2 |
| 258 | F | 81 | 70 | 1.6 | 27.3 | 2 |
| 304 | F | 88 | 70 | 1.62 | 26.7 | 2 |
| 281 | F | 64 | 79 | 1.58 | 31.6 | 3 |
| 273 | F | 76 | 80 | 1.59 | 31.6 | 3 |
| 368 | F | 68 | 99.8 | 1.61 | 38.6 | 3 |
| 140 | M | 81.9 | 73.0 | 1.78 | 23.0 | 1 |
| 141 | M | 82.5 | 69.0 | 1.83 | 20.6 | 1 |
| 153 | M | 77.2 | 63.0 | 1.66 | 22.9 | 1 |
| 154 | M | 47.8 | 69.0 | 1.80 | 21.3 | 1 |
| 155 | M | 72.4 | 76.2 | 1.75 | 24.9 | 1 |
| 159 | M | 89.3 | 75 | 1.82 | 22.6 | 1 |
| 298 | M | 69 | 78 | 1.81 | 23.9 | 1 |
| 317 | M | 81 | 74 | 1.77 | 23.8 | 1 |
| 16 | M | 78.9 | 79.0 | 1.70 | 27.3 | 2 |
| 18 | M | 85.5 | 79.8 | 1.70 | 27.6 | 2 |
| 22 | M | 84.3 | 80.0 | 1.70 | 27.7 | 2 |
| 147 | M | 82.2 | 82.0 | 1.70 | 28.4 | 2 |
| 148 | M | 73.9 | 80.6 | 1.74 | 26.6 | 2 |
| 156 | M | 75.8 | 88.55 | 1.83 | 26.4 | 2 |
| 160 | M | 83.1 | 70.9 | 1.68 | 25.1 | 2 |
| 167 | M | 68.9 | 81.7 | 1.78 | 25.8 | 2 |
| 270 | M | 76 | 83 | 1.71 | 28.4 | 2 |
| 285 | M | 69 | 91 | 1.77 | 29.2 | 2 |
| 307 | M | 75 | 81 | 1.65 | 29.8 | 2 |
| 12 | M | 75.5 | 101.0 | 1.79 | 31.5 | 3 |
| 13 | M | 62.7 | 93.0 | 1.75 | 30.4 | 3 |
| 15 | M | 67.4 | 99.0 | 1.77 | 31.6 | 3 |
| 120 | M | 55.6 | 105.6 | 1.72 | 35.7 | 3 |
| 122 | M | 68.6 | 127.0 | 1.88 | 35.9 | 3 |
| 124 | M | 76.2 | 93.4 | 1.70 | 32.3 | 3 |
| 142 | M | 75.8 | 118.0 | 1.78 | 37.2 | 3 |
| 143 | M | 71.6 | 77.0 | 1.60 | 30.1 | 3 |
| 144 | M | 78.5 | 95.5 | 1.73 | 31.9 | 3 |
| 145 | M | 77.3 | 100.0 | 1.74 | 33.0 | 3 |
| 151 | M | 77.1 | 92.2 | 1.65 | 33.9 | 3 |
| 166 | M | 72.4 | 90.6 | 1.71 | 31.0 | 3 |
| 261 | M | 73 | 103.6 | 1.80 | 32 | 3 |
| 271 | M | 74 | 92 | 1.67 | 32.8 | 3 |
| 299 | M | 72 | 111 | 1.72 | 37.7 | 3 |

**Supplementary Table 2. Statistics corresponding to CLAMS comparison of 8-week-old male and female mice to 18-week-old mice on STD or HFD.** Comparisons were made using ANCOVA in CalR with weight considered a dependent variable.

|  | **Full Day** | **Light** | **Dark** |
| --- | --- | --- | --- |
|  | **Group Effect** | **Group Effect** | **Group Effect** |
| **8-week-old Male vs 8-week-old Female** | | | |
| Hourly Food Consumed (kcal) | 0.0643 | 0.0060 ** | 0.1547 |
| Total Food Consumed (kcal) | 0.0492 * | 0.0744 | 0.0514 |
| Oxygen Consumption (ml/hr) | 0.1499 | <0.001 *** | 0.1238 |
| Carbon Dioxide Production (ml/hr) | <0.001 *** | <0.001 *** | 0.1621 |
| Energy Expenditure (kcal/hr) | <0.001 *** | <0.001 *** | <0.001 *** |
| Respiratory exchange Ratio (RER) | 0.1257 | 0.4162 | 0.0444 * |
| Locomotor Activity (beam breaks) | 0.1432 | 0.2093 | 0.1116 |
| Ambulatory Activity (beam breaks) | 0.1131 | 0.2180 | 0.0770 |
| **8-week-old Male vs Male STD** | | | |
| Hourly Food Consumed (kcal) | 0.5260 | 0.9356 | 0.0144 * |
| Total Food Consumed (kcal) | 0.9025 | 0.7565 | 0.7414 |
| Oxygen Consumption (ml/hr) | 0.0182 * | 0.0206 * | 0.0242 * |
| Carbon Dioxide Production (ml/hr) | <0.001 *** | <0.001 *** | <0.001 *** |
| Energy Expenditure (kcal/hr) | 0.0085 ** | 0.0157 * | 0.0079 ** |
| Respiratory exchange Ratio (RER) | <0.001 *** | 0.0029 ** | <0.001 *** |
| Locomotor Activity (beam breaks) | 0.0907 | 0.1035 | 0.0858 |
| Ambulatory Activity (beam breaks) | 0.1214 | 0.1255 | 0.1242 |
| **8-week-old Male vs Male HFD** | | | |
| Hourly Food Consumed (kcal) | 0.9837 | 0.3533 | 0.6399 |
| Total Food Consumed (kcal) | 0.5589 | 0.3711 | 0.6641 |
| Oxygen Consumption (ml/hr) | 0.8531 | 0.5989 | 0.8910 |
| Carbon Dioxide Production (ml/hr) | 0.8531 | 0.3361 | 0.8511 |
| Energy Expenditure (kcal/hr) | 0.7746 | 0.5835 | 0.9833 |
| Respiratory exchange Ratio (RER) | <0.001 *** | <0.001 *** | <0.001 *** |
| Locomotor Activity (beam breaks) | 0.8935 | 0.8670 | 0.9142 |
| Ambulatory Activity (beam breaks) | 0.7402 | 0.6916 | 0.7817 |
| **8-week-old Female vs Female STD** | | | |
| Hourly Food Consumed (kcal) | 0.0561 | 0.0227 * | 0.1094 |
| Total Food Consumed (kcal) | 0.0451 * | 0.1048 | 0.0401 * |
| Oxygen Consumption (ml/hr) | 0.7274 | 0.8209 | 0.6400 |
| Carbon Dioxide Production (ml/hr) | 0.8051 | 0.8717 | 0.7954 |
| Energy Expenditure (kcal/hr) | 0.7389 | 0.8675 | 0.6216 |
| Respiratory exchange Ratio (RER) | 0.0518 | 0.1685 | 0.0360 * |
| Locomotor Activity (beam breaks) | 0.6159 | 0.5210 | 0.6900 |
| Ambulatory Activity (beam breaks) | 0.9756 | 0.8529 | 0.9397 |
| **8-week-old Female vs Female HFD** | | | |
| Hourly Food Consumed (kcal) | 0.9568 | 0.6960 | 0.7974 |
| Total Food Consumed (kcal) | 0.9168 | 0.9361 | 0.8761 |
| Oxygen Consumption (ml/hr) | <0.001 *** | <0.001 *** | 0.0027 ** |
| Carbon Dioxide Production (ml/hr) | 0.6521 | 0.0950 | 0.5375 |
| Energy Expenditure (kcal/hr) | 0.0036 ** | 0.0012 ** | 0.0113 * |
| Respiratory exchange Ratio (RER) | <0.001 *** | <0.001 *** | <0.001 *** |
| Locomotor Activity (beam breaks) | 0.5628 | 0.5210 | 0.5972 |
| Ambulatory Activity (beam breaks) | 0.8795 | 0.8782 | 0.8888 |
| **Male STD vs Female STD** | | | |
| Hourly Food Consumed (kcal) | 0.4264 | 0.9884 | 0.2227 |
| Total Food Consumed (kcal) | 0.6648 | 0.6502 | 0.7288 |
| Oxygen Consumption (ml/hr) | 0.0328 * | 0.0500 * | 0.0289 * |
| Carbon Dioxide Production (ml/hr) | 0.0011 ** | 0.0016 ** | 0.0057 ** |
| Energy Expenditure (kcal/hr) | 0.0498 * | 0.0123 * | 0.0380 * |
| Respiratory exchange Ratio (RER) | 0.5126 | 0.3538 | 0.7114 |
| Locomotor Activity (beam breaks) | 0.4184 | 0.3503 | 0.4638 |
| Ambulatory Activity (beam breaks) | 0.7263 | 0.5185 | 0.8602 |
| **Male HFD vs Female HFD** | | | |
| Hourly Food Consumed (kcal) | 0.4363 | 0.4146 | 0.0321 * |
| Total Food Consumed (kcal) | 0.3200 | 0.3224 | 0.3204 |
| Oxygen Consumption (ml/hr) | 0.1263 | 0.1929 | 0.0976 |
| Carbon Dioxide Production (ml/hr) | 0.2201 | 0.4143 | 0.1256 |
| Energy Expenditure (kcal/hr) | 0.1288 | 0.2143 | 0.0935 |
| Respiratory exchange Ratio (RER) | 0.0912 | 0.1514 | 0.0610 |
| Locomotor Activity (beam breaks) | <0.001 *** | <0.001 *** | <0.001 *** |
| Ambulatory Activity (beam breaks) | 0.0011 ** | 0.0054 ** | 0.0015 ** |
| **Male STD vs Male HFD** | | | |
| Hourly Food Consumed (kcal) | 0.0535 | 0.3422 | 0.0141* |
| Total Food Consumed (kcal) | 0.3958 | 0.3916 | 0.3920 |
| Oxygen Consumption (ml/hr) | 0.9997 | 0.8502 | 0.0431 * |
| Carbon Dioxide Production (ml/hr) | 0.0062 ** | 0.0200 * | 0.0034 ** |
| Energy Expenditure (kcal/hr) | 0.0447 * | 0.8423 | 0.0261 * |
| Respiratory exchange Ratio (RER) | <0.001 *** | <0.001 *** | <0.001 *** |
| Locomotor Activity (beam breaks) | 0.1209 | 0.1017 | 0.1350 |
| Ambulatory Activity (beam breaks) | 0.0967 | 0.0623 | 0.1291 |
| **Female STD vs Female HFD** | | | |
| Hourly Food Consumed (kcal) | 0.2733 | 0.3904 | 0.2825 |
| Total Food Consumed (kcal) | 0.2677 | 0.5047 | 0.1981 |
| Oxygen Consumption (ml/hr) | 0.1461 | 0.0419 * | 0.3934 |
| Carbon Dioxide Production (ml/hr) | 0.3672 | 0.7817 | 0.0555 |
| Energy Expenditure (kcal/hr) | 0.3197 | 0.0912 | 0.7864 |
| Respiratory exchange Ratio (RER) | <0.001 *** | <0.001 *** | <0.001 *** |
| Locomotor Activity (beam breaks) | 0.9850 | 0.6752 | 0.8697 |
| Ambulatory Activity (beam breaks) | 0.9301 | 0.8702 | 0.8072 |
